# Preparedness to face the COVID-19 pandemic – is India missing the mark?

**DOI:** 10.7189/jogh.10.020338

**Published:** 2020-12

**Authors:** Devasahayam J Christopher, Barney TJ Isaac, Balamugesh Thangakunam

**Affiliations:** Department of Pulmonary Medicine, Christian Medical College, Vellore, India

The Corona Virus Disease (COVID-19) caused by SARS Co-V 2 in late 2019 has resulted in a pandemic infecting more than 8 million across the world with nearly half a million lives already lost. In Wuhan, the epicentre of the disease in China and in Singapore, after an apparent suppression of the pandemic, there has been a rebound of cases. India, despite being on lock down for more than two months witnessed a rise in cases, and this is likely to dramatically worsen as lock down has been released. As part of the preparation for the management of COVID-19 cases, efforts are in full swing and the need to augment the invasive mechanical ventilation (IMV) capacity seems to be a part of the strategy. It is clear that only around 5% of those infected will require critical care and a proportion of them may end up on IMV. More importantly, the outcome in those ventilated is poor- 60% mortality in the best case scenario and as bad as 97% in the worst. IMV is expensive and requires skilled manpower, which cannot be created in a short span of time. On the other hand, 14% of the COVID-19 patients may require admission and only good supportive care, primarily oxygen therapy and non-invasive ventilation. Appropriate preparation to provide good supportive care is crucial. This could avert or reduce the need for IMV and is likely to save more lives. Such a model of cost-effective care is more appropriate for India and this may be something that other resource limited countries could emulate.

## REBOUND OF CASES

According to John Hopkin’s Corona virus resource centre the number of confirmed cases with COVID-19 was 8 079 076 with 438 171 deaths as of 16th June 2020 [1]. A variety of measures are being taken by countries around the world to slow the spread of the disease. As of 21st April 2020 more than a third of the planet's population is under some form of restriction [2]. However the cases and deaths are continuing to rise in most countries despite the lock down. Mathematical modelling has predicted that sustained lockdown or repeated lockdown is going to be required to prevent rebound of infections [3]. The lockdown in India which commenced on 24th March 2020, had been effective in blunting the exponential growth to a linear growth. After a few rounds of extensions, the lockdown was released by the end of May in a phased manner. This has already resulted in a rebound of cases and lockdowns are again being considered in selected hotspots of the country.

## THE HEALTH CARE NEEDS AND CAPACITY

Based on the current trend, a team of modelling experts convened under the leadership of the principal scientific advisor to the government of India have modelled the likely scenario weeks down the line, using a statistical model ‘COVID-19 Med Inventory’ [4]. The prediction is that by mid-July, there could be a steep increase in the cases in India to 30,49 000 with 79 000 requiring ICU beds, 55 000 requiring ventilators and 5700 dying every day. This situation is likely to worsen further.

The Indian health ministry has revealed [5] that there are 15 980 isolation beds and 37 326 quarantine beds for the care of COVID-19 patients. The Indian Society of Critical Care Medicine, estimates that there could at best be 100 000 ICU beds and between 35 000 and 45 000 ventilators in the country (mostly in the private sector, and out of reach of the vast majority of the population) [6]. Since these beds would be required to manage a variety of critically ill medical and surgical patients, it is not clear how much could be set apart for the care of COVID-19 patients.

An empowered committee of the ministry of health has asked the senior industry leaders to amplify the logistics supply chain for ventilators and PPEs. They have appealed to both the public and private sector [7]. Adding to the shortage of equipment, skilled workforce is required to manage these patients, and India would fall woefully short [6].

## OUTCOMES WITH INVASIVE MECHANICAL VENTILATORY SUPPORT

As India prepares to address the imminent escalation of health care needs from this pandemic, the aim would be to save maximum number of lives. It is important to understand the cost-benefit ratios of the various interventions, in order to make optimal use of the limited resources. In nearly 81% of symptomatic patients, the disease is mild, and these individuals are likely to get better without any specific measures [8]. Around 14% may have moderate symptoms and need hospitalisation due to hypoxia but not intensive care unit (ICU) care and an additional 5% may require care in an ICU, due to multiorgan failure or refractory hypoxia [8]. The need for invasive mechanical ventilation (IMV) in those who are critically ill is high ranging from 42 to 100 percent in various published cohorts [9].

The outcome of patients who needed invasive ventilation has been abysmal. In the initial report from 552 hospitals across China[10], the mortality rate of those on IMV was 60% (assuming all who died in hospital were ventilated); this is despite one fifth of them having received ECMO. In a subsequent report from China, the mortality among those on IMV was 97% [11]. A large cohort [12] of 5700 hospitalised patients from New York City area reported 88.1% mortality among those who received IMV.

## GOOD SUPPORTIVE CARE IS THE WAY FORWARD

Although several large randomized drug trials are under way, current survival from severe COVID-19 depends entirely on providing the best possible supportive care [13]. It is amply clear that only a small proportion of patients require invasive ventilation, who despite this treatment have poor outcomes. Invasive ventilation is resource intensive and requires a highly skilled team, which cannot be created at short notice. Therefore, in resource limited settings, it would be more fruitful and many more lives may be saved by focussing on good supportive care, which would result in less patients needing IMV. All health care providers who monitor asymptomatic/mildly symptomatic patients or the patients themselves should use a pulse oximeter, which is economical and easily available to assess desaturation and thus the need for hospitalisation. As a rule of thumb, adults with room air oxygen saturation ≤93% [8] could be considered for hospitalisation.

Providing supplemental oxygen is central to the care of hospitalised patients. In 2015, the Lancet Commission on Global Surgery revealed that approximately one-quarter of hospitals surveyed in resource-limited countries lack sufficient oxygen supply [14]. Thus hospitals being prepared to care for COVID-19 patients requiring hospital admission should identify sources for procuring high-quality medical grade oxygen. Common oxygen sources are oxygen generating plants, liquid oxygen storage tanks, oxygen cylinder and oxygen concentrator. The total oxygen requirement for a hospital can be calculated using the WHO essential resource planning tool. There should be a well-defined plan to boost oxygen delivery capacity in case there is a surge of patients with COVID-19 requiring oxygenation [15].

**Figure:**
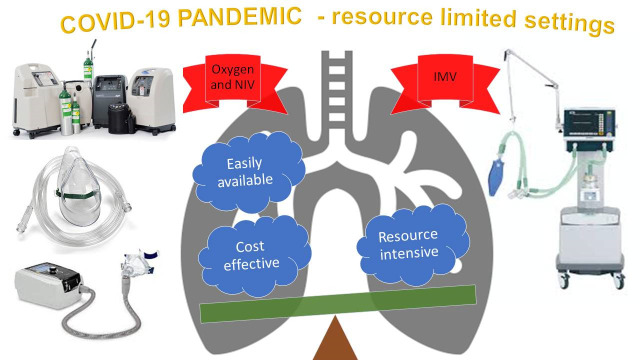
Photo: from the authors’ own collection, used with permission.

Oxygen cylinders are most frequently used mode and these are purchased by small hospitals and clinics for their requirements. They are bulky and transporting cylinders for refilling is labour intensive. Bed side oxygen concentrators are very helpful plug-and-use devices which can be easily operated with minimum training. Most of the current concentrators can deliver up to 6 lit/min oxygen flow and may be enough for moderately hypoxic patients with COVID-19. In case higher flows are required multiple concentrators can be serially connected. Effort should be taken to scale up the availability of concentrators. They are more important and useful for a greater number of patients than invasive ventilators and far less expensive.

In resource limited settings where invasive ventilators cannot be used (because they are not available, or expertise is not available) simple and less costly equipment which are easier to use (like non-invasive ventilation (NIV) or CPAP) can be used [13]. Continuous positive airway pressure (CPAP) is now being advocated by some physicians who are managing COVID-19 patients. It could prevent or delay the need for invasive ventilation. CPAP is commonly used in the treatment of obstructive sleep apnea and these devices are available at a modest cost. This can be combined with additional oxygen, either entrained to the device or given through small bore nasal prongs. It is relatively simple to use and it is not difficult to teach physicians caring for COVID-19 patients the operation of this machine. However, to prevent dispersion of exhaled air a viral filter needs to be introduced in the circuit. Prone ventilation has been advocated in the management of patients not only on IMV but also on NIV/CPAP or just on oxygen supplementation if they do not have any contraindication [16]. This does not involve much cost and could facilitate the oxygenation of these patients by reducing the VQ mismatch, recruitment of posterior lung segments, decrease shunting and augmenting secretion clearance [16]. Use of CPAP with prone positioning may be a good strategy to defer or avoid IMV.

## CONCLUSION

In preparation for the imminent escalating health care needs of the COVID-19 pandemic, various strategies are being put in place. Augmentation of bed strength and apportioning beds specifically for care of COVID-19 patients is crucial and a challenge India may struggle to achieve if the predicted increase of cases becomes a reality, on account of poor public health care system and resource constraints. Undue emphasis has been placed on procurement of additional ventilators, which would deplete the available resources and provide very little additional returns. We feel prompt identification of hypoxia by careful monitoring, providing good supportive care for hospitalised patients with infrastructure to provide supplemental Oxygen and optimal use of Non-invasive ventilatory modes to defer or delay IMV, will save more lives. This is a model India can create and pass on to other resource-limited countries.
